# Nine-year-long complex humeral nonunion salvaged by distraction osteogenesis technique: a case report and review of the literature

**DOI:** 10.1186/s12893-022-01524-z

**Published:** 2022-03-03

**Authors:** Qiyu Jia, Yanshi Liu, Abudusalamu Alimujiang, Jian Guo, Dongsheng Chen, Yingbo Wang, Aihemaitijiang Yusufu, Chuang Ma

**Affiliations:** 1 Department of Microrepair and Reconstruction, The First Affiliated Hospital of Xinjiang Medical University, Urumqi, Xinjiang China; 2 College of Chemical Engineering, Xinjiang Normal University, Urumqi, Xinjiang China

**Keywords:** Humeral nonunion, Distraction osteogenesis, Radial nerve palsy, Tendon transfers, Case report

## Abstract

**Background:**

Humeral nonunion with significant bone loss or shortening is uncommon and poses a complex clinical problem. We present a case of humeral nonunion with a large segmental bone defect treated with the distraction osteogenesis technique and remedy the radial nerve palsy produced during distraction osteogenesis by forearm tendon transfers. The reconstruction of upper limb function was achieved with satisfactory results. This case provides a referenceable alternative method for repairing large segmental bone defects due to complex nonunion of the upper extremity, as well as a remedy in the unfortunate event of radial nerve palsy, providing a reference and lessons learned for the treatment of similar cases and the management of possible complications.

**Case presentation:**

A 31-year-old male patient experienced 9 years of hypertrophic nonunion due to an unreliable internal fixation. The radiographs showed the absence of bone bridging between the two fragments, loosening of the screws, and extensive osteolysis around the internal screws. The patient was treated with distraction osteogenesis. At the end of the distraction period, the patient unfortunately developed right radial nerve paresis, which was salvaged by forearm tendon transplantation, and finally reconstructed hand function and achieved bone union of the humerus.

**Conclusion:**

Distraction osteogenesis, although not a panacea for all humeral nonunions with significant segmental bone loss, does offer a viable salvage procedure in this unusual and often complex clinical problem. When irreversible radial nerve palsy occurs during distraction, forearm tendon transfers can have a good clinical effect.

## Background

Humeral nonunion is estimated to account for 2 to 10% of surgically treated patients [1–3]. Nonunion humeral shaft fractures often lead to pain, prolonged disability leading to reoperation, long-term absence from work, and impaired quality of life. An increased incidence of nonunion is associated with many factors, including smoking, abusing alcohol, using anti-inflammatory drugs, unstable fixation, poor patient compliance, devitalization of soft tissues, insufficient immobilization, and infection [4, 5].

Reconstruction of humeral shaft nonunions is a challenge for orthopedic surgeons, especially for osteoporosis, bone defects, and infection [6, 7]. Several strategies have been proposed to resolve these complex problems, including (double) plating augmented with autologous bone grafts, locking intramedullary nails, unilateral external fixators, and circular external fixation [8–12].

A patient with distal humerus nonunion for nine years is described in the present study. A plate was implanted for internal fixation as preliminary management. Unfortunately, unstable fixation led to hypertrophic nonunion, resulting in bone resorption around the screws and a large bone defect. Ultimately, the injured extremity was salvaged successfully by distraction osteogenesis, forearm tendon transfers remedied a series of symptoms of radial nerve palsy caused by distraction osteogenesis, and satisfactory clinical outcomes were finally achieved.

## Case presentation

**Fig. 1:**
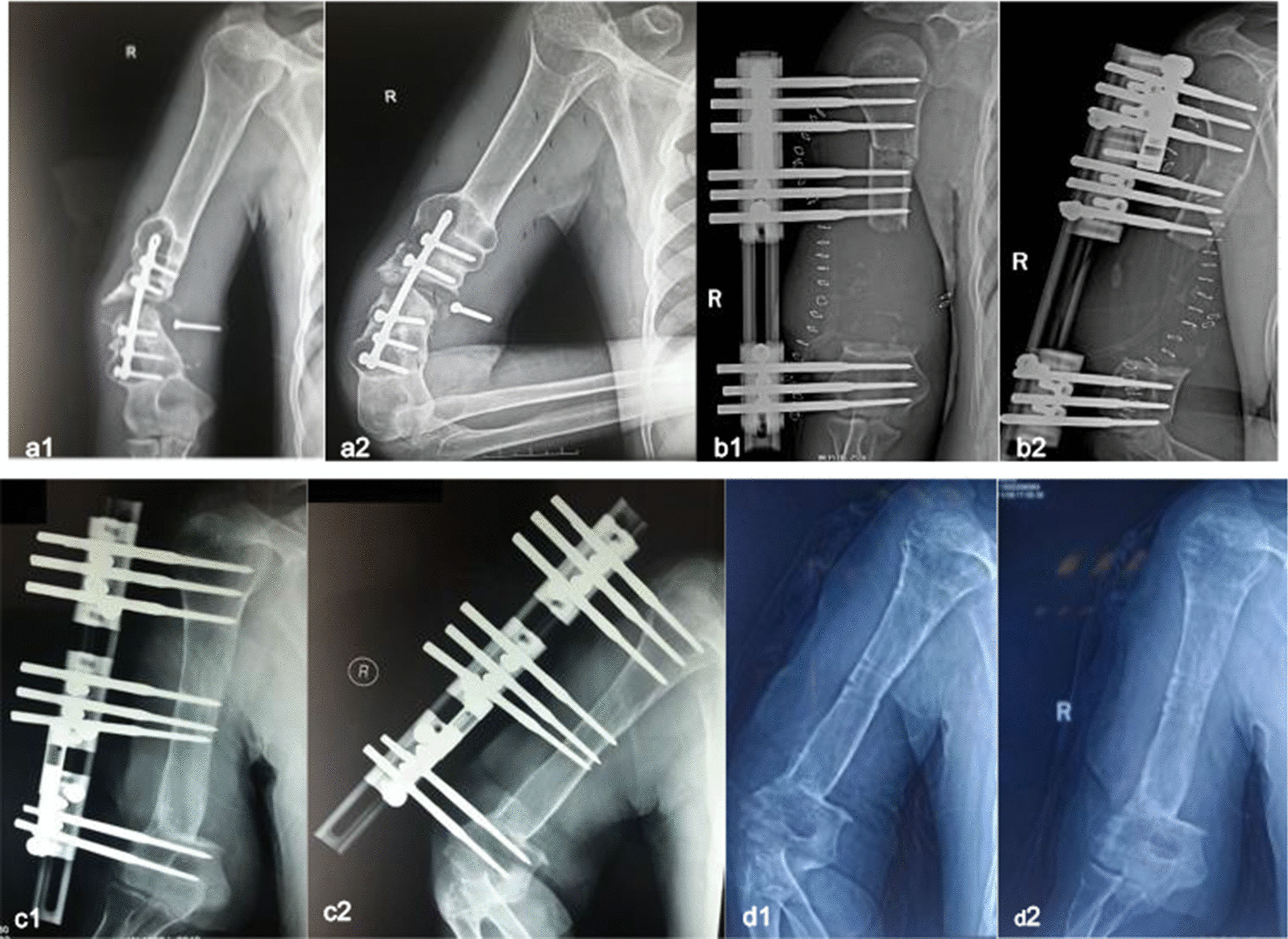
Radiographs of this patient at different stages of distraction osteogenesis treatment. **a1**, **a2** Preoperative. **b1**, **b2** Postoperative. **c1**, **c2** 8 months postoperatively. **d1**, **d2** 12 months after removal of the external fixator

A 31-year-old man presented with nonunion of the right humeral diaphysis. He sustained a closed fracture nine years ago due to a fall from a height and was treated with internal fixation at a local hospital. The patient’s condition improved, was discharged and did not return to the hospital for a clinical follow-up. Two years ago, the patient suddenly had abnormal motion in the right upper arm, accompanied by pain, while performing daily living activities. The patient did not go to the hospital for examination and treatment due to personal reasons. The pain symptoms were gradually relieved after using over-the-counter pain relievers. However, the patient continued to experience abnormal activity of the right upper arm, and experienced obvious pain during strenuous activities. Twenty days ago, the patient developed active protrusions on the inner skin of the right upper arm, accompanied by obvious pain, and came to our department for help. The patient complained of moderate pain at the nonunion site that worsened with stress, in addition to pathological barriers to motion and severe functional disability of the injured limb. The radiographs showed the absence of bone bridging between the two fragments, loosening of the screws, and extensive osteolysis around the internal screws (Fig. 1a). The diagnosis was considered to be humeral nonunion.

**Fig. 2:**
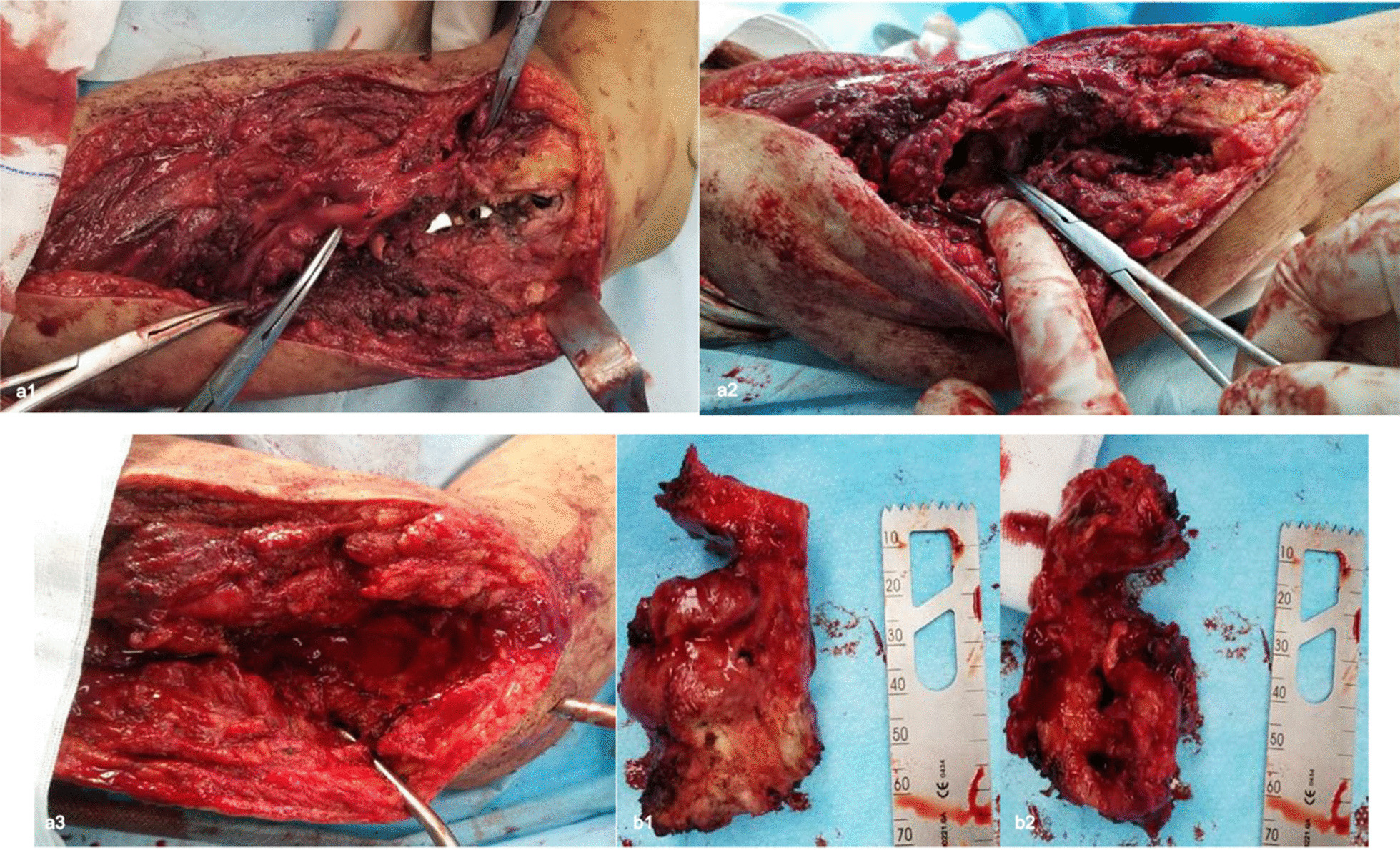
Intraoperative pictures of the affected area. **a1** Hypertrophied bone encapsulating the radial nerve. **a2** Disorganized bone structure. **a3** Bone defect area after amputation of abnormal hypertrophy. **b** Hypertrophic bones **b1** and **b2** were removed intraoperatively

The operation was performed under general anesthesia. Serious scar adhesion and chaotic local anatomy were observed after a full-length incision on the lateral side of the right humerus. We separated the subcutaneous tissue and explored the radial nerve, which was dark in color and complete in continuity. The radial nerve was carefully separated from the proximal end to the distal end. It was wrapped by abnormal hyperplasia of bone 5 cm above the lateral condyle of the humerus, and there was local compression (Fig. 2a1). The hypertrophic bone callus was carefully excised using a rongeur, and the radial nerve was released and protected by rubber strip traction. When the periosteum was separated to depth, a large amount of callus was observed between the fracture gaps, but the cortical bone was uneven (Fig. 2a2). Part of the cortical bone was removed, the plate was found to be wrapped by the bone of the hollow structure, and the screw and plate were loosened. The bone around the implant was removed, and the plate and screw were removed. The distal humerus and the elbow joint cavity were connected. The bones in the affected area of the humerus were all sclerotic bones, and there was no medullary cavity or poor blood supply. Radical debridement was performed to reopen the intramedullary canal, including bone decortication, excision of fibrous tissue and ablation of necrotic bone. Under the image intensifier, 8–9 parallel pins were inserted at the lateral side of the humerus, perpendicular to the anatomical axis. Three pins were fixed at the proximal and distal ends of the humerus. The other three pins were inserted into the transported bone segment. All pins were confirmed to be parallel to each other in the same plane. A minimally invasive subperiosteal osteotomy was performed between the two sets of pins at the proximal end of the humerus (Fig. 2a3, b).

The lengthening procedure was initiated on the seventh postoperative day. Based on the patient's pain tolerance, a 120-day lengthening period allowed for 8.5-cm lengthening (Fig. 1b), a rate of 0.71 mm/day followed by eight months of a consolidation phase (Fig. 1c). Eventually, the bone completely healed. The external fixation index (EFI) was 43.18 days/cm for this patient.

**Fig. 3:**
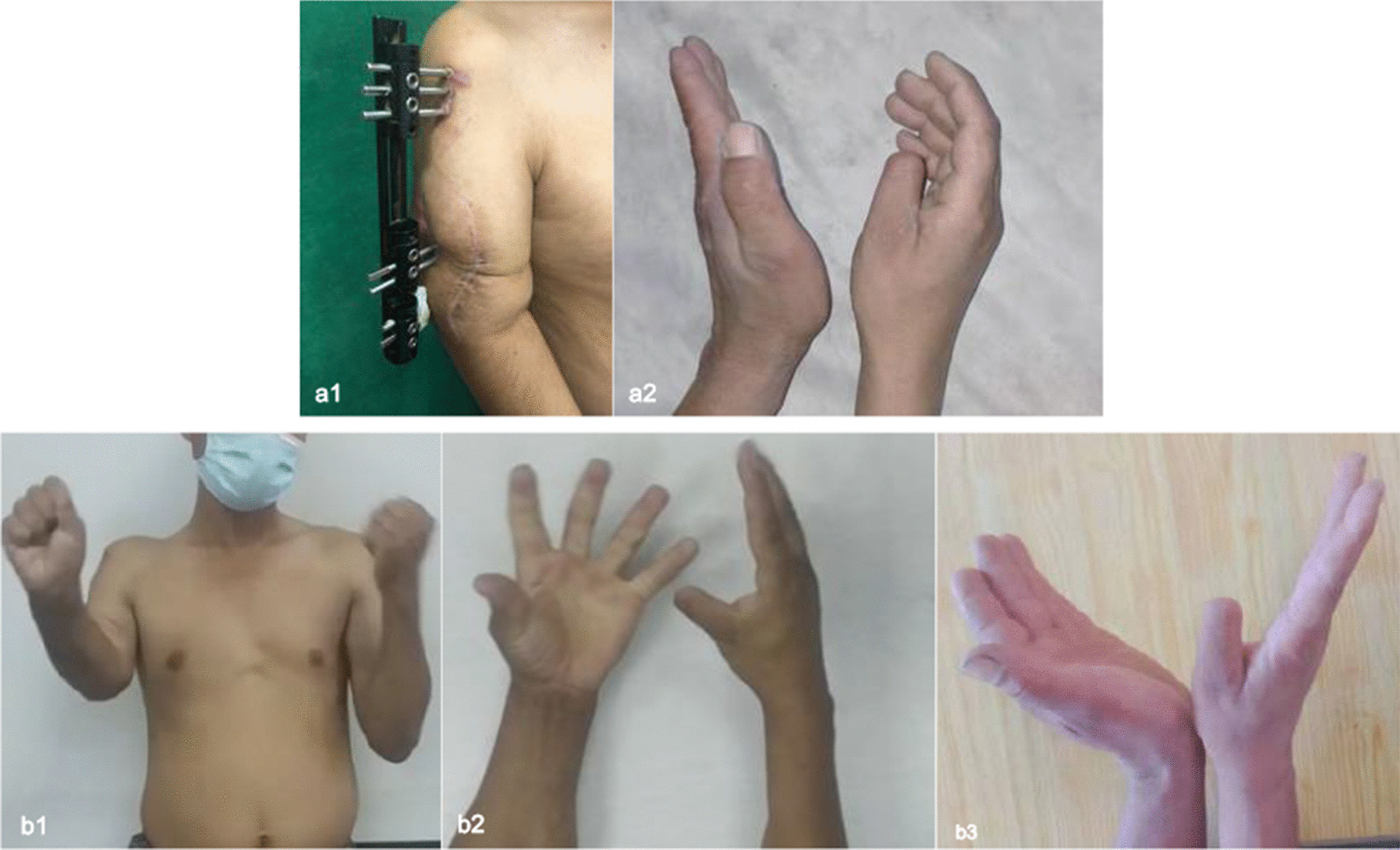
The patient showed radial nerve paresis at the end of distraction osteogenesis. **a1** Muscle buildup around the distal humerus at the end of distraction. **a2** The right hand showed wrist extension, finger extension, and thumb extension dysfunction. **b1**, **b2** Radial nerve function was reconstructed after forearm tendon transfers

This patient presented with radial nerve palsy with a wrist drop and obstructed thumb and finger extension 135 days after the surgery (1 week after the distraction phase was completed at the first clinical visit) (Fig. 3a2). Additional surgical interventions were performed to manage radial nerve palsy.

A lateral incision of the right upper arm was made to delicately separate the tissue layer-by-layer under the microscope, avoiding the brachial artery. The brachial artery was buried in the scar tissue and then completely released. Serious incarceration and degeneration of the radial nerve caused by the surrounding soft tissues at the lengthening of the lower humerus could be observed. The proximal radial nerve lost its normal shape in a large area. Neural anastomosis and transplantation could not be performed during intraoperative evaluation, and right forearm tendon transplantation was therefore conducted to reconstruct back extension function.

A longitudinal incision was made at the junction of the middle and upper third of the radial side of the right forearm and entered from between the brachioradialis and the extensor carpi radialis longus to locate and cut the pronator teres (PT), displace and anastomose it to the extensor carpi radialis brevis (ECRB). Next, the flexor carpi ulnaris (FCU) was found and cut, in which the FCU was anastomosed to the extensor digitorum cornmunis (EDC) around the ulnar border of the forearm. At the same time, the extensor pollicis longus (EPL) was severed at the musculotendinous junction, drawn out at the metacarpophalangeal joint of the thumb, and rerouted to the palmaris longus (PL) through the tunnel of the abductor pollicis longus.

After docking, the patient was encouraged to perform progressive functional exercises. One month after the end of the consolidation period, sufficient consolidation in the distraction zone and complete union at the docking site was determined, and the external fixator was removed. At the same time, the patient had fair wrist extension activity, grade 3 muscle strength, and grade 4 muscle strength for flexion.

At the last clinical visit (12 months after frame removal), the patient had good wrist extension activity, good thumb extension and finger extension function (Fig. 3b). Elbow range of motion was slightly limited (10° to 90°), but based on the Mayo criteria, was subclassified as good, and hand grip strength increased without significant difficulty in performing daily living activities.

## Discussion and conclusions

Humeral nonunion with significant bone loss or shortening is an uncommon and complex clinical problem [13]. The etiology includes bone loss due to open fractures, infection, tumor resection, and bone resorption associated with failed internal fixation [13–15]. Although the methods to address humeral nonunion have significantly improved, the surgeon performing the procedure will experience challenges in the clinical setting regarding occasional complex cases.

Achieving biomechanical stability is a reasonable method for treating nonunion humeral fractures. Compression plates, which are now widely used, are not always sufficiently stable unless the soft tissue surrounding the fracture is widely incised, especially when osteopenia is present or previous attempts at fixation have failed. In addition, this method is not without complications, the most important of which is damage to the radial nerve (3 to 29%) [16], as at least six cortical screws are fixed on either side of the nonunion [8, 14]. Reconstruction with two plates at right angles has been advocated to increase local stability, but there were no significant differences in clinical outcomes between single- and double-plate constructions [17]. This technique is not recommended for nonunions with infection, osteoporotic bone, long spiral, or large segmented fracture lines, especially distal metaphyseal nonunions, as more than six cortices or augmentation with strut grafts are required [16]. In this case, nonunion of the distal humerus was not suitable for acute shortening due to the large bone defect area, and there was insufficient space to engage six cortices. If it was decided that osteotomy would not be performed, it was clear that the hollow structure of the hypertrophied bone would not provide favorable support for the plate.

Intramedullary locking nails are controversial in the treatment of humeral nonunions [18]. Unlike lower limb nonunion, lack of weight bearing status and inadequate compression diminish the success rate in humeral shaft nonunions [19]. Autologous bone grafting is osteogenic, osteoinductive, and osteoconductive. However, the traditional supply of autogenous materials may be insufficient when the bone defect in the nonunion area is large. Fibula transplantation is also a good choice when there are segmental defects in humeral nonunion. Heitmann et al. described 15 patients with segmental defects who were treated with an osteoseptocutaneous fibular transplant and showed good functional and cosmetic results. This technique is, however, complex and associated with a high rate of complications.

External fixation conserves the soft-tissue envelope and the vitality of the remaining bone [8]. This technique is less invasive and can minimize surgical damage to the fracture. The fixator provides gradual compression to the nonunion site, mimicking the weight-bearing status of the lower limbs [20]. In addition, in the process of fixation, the external fixator can evenly distribute the force on the bone section; effectively eliminate the shear, torsion, and displacement of the fracture end that affects the healing of the fracture; and can adjust the pressure after the operation to achieve lengthening and compression at the same time.

As mentioned earlier, this patient had extensive osteolysis around the implant plate and abnormal callus hyperplasia caused by unstable internal fixation. Bone is a mechanosensitive organ that can respond to the introduction of mechanical stimuli. When high strain forces are present at the fracture site, i.e., inadequate stability, fibrous tissue will remain, and a stable bony callus cannot form. If this condition persists for a long period of time, fibrous nonunion will develop [21]. To date, many mechanical factors that influence fracture healing have been identified, including the magnitude and direction of interfragmentary movement (IFM), the type of fracture, and the geometry of the fracture [22–24]. Notably, when the IFM exceeds a critical level, the vessels are repeatedly disrupted and unable to form at the fracture site, thus preventing the development of stable tissue [25]. A certain amount of mechanical instability can lead to greater IFM and thus increase the risk of nonunion. Severe bone resorption was present in this patient. Previous pathological studies revealed severe inflammatory reactions around failed orthopedic implants [26, 27]. Unstable internal fixation inevitably causes long-term tissue damage, which leads to a continuous progression of inflammation [28]. This will eventually lead to increased osteoclast production and overactivation [29], inducing the development of bone resorption around the internal fixation. We elected to remove the original plate and remove the surrounding abnormal bone. Then, the upper and middle sections of the humerus were osteotomized, and unilateral external fixator bone lengthening was performed.

Most investigators believe that limb length discrepancy is not a major problem in the upper limb, and lengthening is only required if the discrepancy exceeds 5 cm [30, 31]. After removing the abnormal bone, our patient had a gap of approximately 8.5 cm. Obviously, acute shortening was not applicable at that time and needed to be treated by means of bone lengthening. The potential for effective lengthening of the humerus was recognized. The incidence of callus formation during distraction was significantly higher than that of the tibia [32]. In the lower extremities, when the lengthening exceeds 20%, the incidence of complications increases, but this does not occur in the humerus [33, 34]. Obviously, this patient was suitable for humerus lengthening.Monolateral external fixation instead of the Ilizarov frame or hybrid fixation was used due to its lower complication rate [35, 36]. In addition, a monorail external fixator was sufficient to achieve lengthening and was more convenient to wear, with less negative impact on the patient’s lifestyle [37].

Iatrogenic nerve injury, a complication related to limb lengthening, poses a challenge for orthopedic surgeon [38], as the nerves are forced to stretch along with other anatomical structures. A nerve might also become compromised as a result of the use of fixation devices placed near the nerve itself [39]. The incidence of peripheral nerve injury after limb lengthening procedures ranges from 3 to 30% [40]. In this case, the symptoms of radial nerve paralysis appeared after the lengthening period. Due to muscle attachment to the transitional bone, with the development of the distraction process, the muscle attached to the transitional bone moves distally together, resulting in the accumulation of muscle around the distal humerus (Fig. 3a1), compressing the radial nerve and resulting in radial nerve palsy.

Radial nerve injuries can permanently limit physical function, which is largely unacceptable to the patient. Functional reconstruction at this point is critical. Indications for radial nerve palsy with tendon grafting include severe injury where neurorrhaphy is not possible, brachial plexus injury, absence of extensor musculo-tendinous units, no clinical and electromyographic signs of recovery 6 months after neurorrhaphy, late presentation (> 10 months) or severe scarring or muscular atrophy. High radial nerve palsy, as demonstrated in this patient, results in paralysis of all extensor muscles, leading to wrist drop. When the injury occurs proximal to the forearm, the Radial nerve palsy can be low. In such cases, wrist dorsiflexion is preserved due to sparing of the extensor carpi radialis longus (ECRL), but the fingers and thumb cannot be extended.

For surgical treatment, triple tendons transfer will be required for high radial nerve palsy, whereas for low radial nerve palsy, restoration of wrist extension may not be required [41]. If the radial nerve is completely unrecoverable, an end-to-end transfer should be performed, which will result in a straight direction of tension, but if recovery of the nerve is still possible, an end-to-side transfer of all tendons should be performed so that the native tendon can resume its function once recovery is achieved [42, 43]. There are various combinations of tendon transfers for radial nerve palsy. The most common are the use of PT transfer to ECRB to reconstruct wrist extension and PL transfer to EPL to restore thumb extension. For reconstruction of finger extension, in addition to FCU, there are also options for Flexor carpi radialis (FCR) and flexor digitorum superficialis (FDS) to dock with EDC. All three of these common transfer modalities have achieved good results in previous clinical treatments.

We adopted forearm tendon transfers to achieve reconstruction of wrist extension, finger extension, and thumb extension and abduction and achieved good results after surgery. Intraoperatively, it is important to note that when the FCU is transferred to the EDC, the four EDC tendons should be divided distal to their musculotendinous junction and woven into the FCU. This should be done near the extensor retinaculum to maintain the smoothest possible glide and to prevent bow-stringing from occurring if the extensor retinaculum is divided. When PL is transferred to EPL, the EPL should then be removed from the third extensor compartment and transposed radially to Lister’s tubercle. This allows for a straighter line of traction from the PL to the EPL. It also provides some additional radial adduction of the thumb in addition to extension. Of course, prior to this unfortunate event, it may have been wise to release the radial nerve in advance to prevent radial nerve palsy when the grafted bone did not reach the docking site during the extension period. The patient was very satisfied with the final overall surgical result and had a much-improved quality of life compared to the previous outcome.

This case demonstrates the successful treatment of a complex humeral nonunion with distraction osteogenesis, providing a referable alternative method for repairing a large segmental bone defect due to similar conditions in the upper extremity, as well as salvage measures in the unfortunate event of radial nerve palsy.

There are several limitations to this case report. First, long-term follow-up results, such as 5 and 10 years, are lacking for this patient. Second, there was no in-depth assessment of the patient's relevant social and psychological functioning indicators.

## Conclusions

Distraction osteogenesis, although not a panacea for all humeral nonunions with significant segmental bone loss, does offer a viable salvage procedure in this unusual and often complex clinical problem. When irreversible radial nerve palsy occurs during distraction osteogenesis, forearm tendon transfers can have a good clinical effect.

## Data Availability

The datasets analyzed during the current study are available from the corresponding author on reasonable request.

## Abbreviations

EFI: External fixation index
IFM: Interfragmentary movement
PT: Pronator teres
ECRB: Extensor carpi radialis brevis
FCU: Flexor carpi ulnaris
EDC: Extensor digitorum cornmunis
EPL: Extensor pollicis longus
PL: Palmaris longus
ECRL: Extensor carpi radialis longus
FCR: Flexor carpi radialis
FDS: Flexor digitorum superficialis
